# The value measurement of emerging therapeutics in renal cell carcinoma: ASCO value framework and ESMO-MCBS

**DOI:** 10.1186/s12913-022-08279-6

**Published:** 2022-07-11

**Authors:** Hyerim Ha, Jin Hyoung Kang, Do Yeun Kim, Seung Jin Bae, Hee Yeon Lee

**Affiliations:** 1 Department of Internal Medicine, Inha University Hospital, Incheon, Republic of Korea; 2 Department of Internal Medicine, Seoul St. Mary’s Hospital, Seoul, Republic of Korea; 3 Department of Internal Medicine, Dongguk University Ilsan Hospital, Goyang, Republic of Korea; 4 College of Pharmacy, Ewha Womans University, Seoul, Republic of Korea; 5 Division of Oncology, Department of Internal Medicine, Yeouido St. Mary’s Hospital, College of Medicine, The Catholic University of Korea, 10, 63-ro, Yeongdeungpo-gu, Seoul, 07345 Republic of Korea

**Keywords:** Values, ASCO value framework, ESMO-MCBS, Carcinoma, renal cell, Tyrosine kinase inhibitor, Immune checkpoint inhibitor

## Abstract

**Purpose:**

Rapid development of novel therapeutics in renal cell carcinoma (RCC) has led to financial burden for patients and society. Value including clinical benefit, toxicity affecting quality of life and cost-effectiveness are a concern, prompting the need for tools to facilitate value assessment of therapeutics. This study reviews the value assessment tools, and evaluates the value of emerging therapeutics in RCC.

**Materials and methods:**

Two medical oncologists used American Society of Clinical Oncology value framework (ASCO VF) v2.0 and European Society for Medical Oncology-magnitude of clinical benefit scale (ESMO-MCBS) v1.1 to phase 3 trials evaluating first-line therapy in patients with metastatic RCC. Follow-up (FU) reports and extended survival data were included. Equivocal aspects and limitations of the tools were discussed.

**Results:**

Six trials (COMPARZ, CheckMate 214, JAVELIN renal 101, Keynote 426, CLEAR, and CheckMate 9ER) were assessed. The control arm was standard-of-care sunitinib in all trials. ASCO VF’s net health benefit, calculated as clinical benefit, toxicity and other bonus point was 11 in pazopanib, 41.9 in nivolumab plus ipilimumab, 22.4 in axitinib plus avelumab, 48.7 in axitinib plus pembrolizumab, 35.2 in lenvatinib plus pembrolizumab, and 50.8 in cabozantinib plus nivolumab. A higher score means a greater treatment benefit. ESMO-MCBS gave grade 5 to nivolumab plus ipilimumab, 4 to pazopanib, lenvatinib plus pembrolizumab and cabozantinib plus nivolumab, 3 to axitinib plus avelumab or pembrolizumab. Both tools had unclear aspects to be applied to clinical practice, and should be more clearly defined, such as endpoint for determining survival benefits or how to standardize quality of life and toxicity.

**Conclusions:**

ASCO VF and ESMO-MCBS were applied to evaluate the newly emerging drugs in RCC and assessed their value. In-depth discussion by experts in various fields is required for appropriate clinical application in a real-world setting.

## Introduction

Annually, an estimated 74,000 people are diagnosed with cancers of kidney and renal pelvis and almost 15,000 people die from these diseases in the Unites States [1]. Kidney cancer is the 10th most common cancer and the 8th most common cause of cancer death [2]. Renal cell carcinoma (RCC) accounts for 85% of kidney cancer and approximately 70% of patients with RCC have clear cell type. The remains consist of papillary, chromophobe and collecting duct tumors [3]. About a tenth of patients with RCC express advanced metastatic disease at the time of diagnosis. About a third of patients who undergo surgical resection for local disease experience recurrence, and need systemic treatment as well [4]. When surgical resection is feasible, it is the treatment of choice in loco-regional RCC. During 1980s and 1990s, cytokine therapy with interferon alpha or interleukin-2 was the systemic treatment available for unresectable or metastatic RCC. Since the 2000s, tyrosine kinase inhibitors (TKIs) and immune checkpoint inhibitors (ICIs) have been shown to be effective in patients with RCC, and various combination therapies have recently been introduced [5, 6]. Although the rapid development of novel therapies has provided insights into the future direction of treatments for RCC, the best choice of drug, and the sequence of drug remain elusive. Currently, due to the rapid development and approval of novel anticancer therapies, the high cost of cancer treatment has become a major concern for patients and the society. The financial toxicity may lead to psychosocial distress, poor quality of life (QoL), and worse patient outcomes. Unfortunately, increased costs do not always correlate with improved patient outcomes. Thus value-based decision-making is critical and includes clinical benefit of the drug, potential toxicities affecting QoL, and the cost-effectiveness [7, 8]. The American Society of Clinical Oncology (ASCO) and the European Society for Medical Oncology (ESMO) independently developed assessment tools to evaluate the value of new anticancer drugs and to facilitate decision-making of patients, doctors and payers [9, 10]. The ASCO value framework (ASCO VF) and ESMO-magnitude of clinical benefit scale (ESMO-MCBS), were released in 2015, ASCO VF was revised in 2016 (v2.0), and ESMO-MCBS in 2017 (v1.1) [11, 12].

This study aims to determine whether value assessment helps clinicians select the right anticancer drugs for RCC and whether they are suitable for social valuation. For the purpose, we review the value assessment tools (ASCO VF and ESMO-MCBS), and evaluate the value of emerging therapeutics in RCC.

## Materials and methods

Phase 3 clinical trials showing positive results of first-line therapy compared with sunitinib in patients with metastatic RCC and approved by United States Food and Drug Administration (US FDA) were evaluated. We searched relevant literature from PubMed and US FDA approval announcements. Two medical oncologist (HH, LH) independently assessed the eligibility of the study. After reviewing US FDA approval announcement and the trials supporting the approval decision, 6 trials were selected. We then retrieved additional data on patient-reported outcomes (PRO) and follow-up (FU) reports via PubMed. Authors applied the ASCO VF v2.0 and the ESMO-MCBS v1.1 to the trials and compared the results. Disagreements between the authors were resolved by consensus based on further discussion. We were trained for the tools and had applied the tools to more than 10 phase 3 trials for previous study. The inter-rater reliability was achieved [13].

### ASCO VF v2.0

The clinical benefit, toxicity score, bonus points, and net health benefit were calculated according to the ASCO VF for advanced disease. In a trial which reported progression-free survival (PFS) and follow up (FU) data with overall survival (OS), the statistically significant OS was selected to determine the clinical benefit score. The toxicity score was evaluated with the main report and supplementary data. Each toxicity was scored according to frequency and grade, but laboratory only toxicities were excluded. Bonus points were scored by the tail of the curve, symptom palliation, QoL, and treatment-free interval. Finally, the net health benefit was calculated based on the clinical benefit score, toxicity score, and bonus points. The monthly cost of drug treatment was estimated according to the body weight of 60 kg. Drug costs only focused on antineoplastic drugs, excluding the co-administered drugs or supportive expenditure in cancer care. We estimated monthly prices for US according to average sales price which reported in drug abacus methodology [14]. The US Department of Veterans Affair Federal Supply Schedule big 4 price which could be access via online was used.

### ESMO-MCBS v1.1

Depending on the primary endpoints of the trials and the survival duration, the evaluation form of ESMO-MCBS was selected. ESMO-MCBS comprises three categories according to treatment goals: adjuvant or curative (form 1), non-curative (form 2) and orphan disease (form 3). The three subtypes in non-curative setting depend on the endpoints (form 2a for OS, form 2b for PFS and form 2c for others). The preliminary grade of clinical benefit was decided by the hazard ration (HR), and the duration of survival gain. The final grade was obtained by adjusting for early stopping or crossover, QoL, toxicity, or plateau of survival curve.

## Results

### Characteristics of trials

In this analysis, among the six phase 3 clinical trials, five trials except pazopanib demonstrated superiority of the drugs over standard-of-care sunitinib as first-line treatment in metastatic RCC. The trial of pazopanib was designed to confirm the non-inferiority. The control arm was sunitinib in the four trials. CheckMate 214 enrolled all patients, while the primary endpoints (OS, PFS, objective response rate (ORR)) were analyzed in International Metastatic RCC Database Consortium (IMDC) intermediate or poor prognostic group. The combination of axitinib and avelumab was analyzed in both programmed death ligand 1 (PD-L1) positive group, and the intention to treat (ITT) population. COMPARZ and CheckMate9ER trials reported QoL data. The summary of the trials is presented in Table 1.

**Table 1:** Summary of clinical trials in RCC

Trial	Treatment	Control	Year	No. of Patients (treatment v. control)	Primary endpoint	Secondary endpoint	Median FU period (months)
COMPARZ	Pazopanib	Sunitinib	2013	1110 (557 v. 553)	PFS	OS, safety, QoL	NA
CheckMate 214	Nivolumab + Ipilimumab	Sunitinib	2018	1096 (550 v. 546)	OS, ORR, PFS (IMDC Intermediate or poor prognostic risk)	OS, ORR, PFS (overall)	25.2
JAVELIN renal 101	Axitinib + Avelumab	Sunitinib	2019	886 (442 v. 444)	OS, PFS (PD-L1+)	PFS (overall), ORR, safety	9.9
Keynote 426	Axitinib + Pembrolizumab	Sunitinib	2019	1062 (432 v. 429)	OS, PFS	ORR	12.8
CLEAR	Lenvatinib + Pembrolizumab	Sunitinib	2021	712 (355 v. 357)	PFS	OS	26.6
CheckMate 9ER	Cabozantinib + Nivolumab	Sunitinib	2021	651 (323 v. 328)	PFS	OS, ORR	18.1

Clinical trials for metastatic RCC included this study were summarized

*FU* follow-up, *PFS* progression-free survival, *OS* overall survival, *QoL* quality of life, *NA* not available, *IMDC* International Metastatic Renal Cell Carcinoma Database Consortium, *PD-L1* programmed death ligand-1

### ASCO value framework

The ASCO VF for advanced disease was applied. All the trials reported HR, thus the clinical benefit score was calculated by HR for death or disease progression. All trials reported improved PFS, and CheckMate 214, Keynote 426, CLEAR and CheckMate 9ER reported improved OS. The COMPARZ trial confirmed non-inferiority of pazopanib, thus omitting the clinical benefit score. In the trial of axitinib plus avelumab, PFS and OS for patients with PD-L1-positive tumor were co-primary endpoints, whereas PFS for ITT population was reported as secondary endpoint, and OS was not reported, yet. US FDA approved axitinib and avelumab in advanced RCC based on improved PFS in ITT populations, regardless of PD-L1 status. Thus, the clinical benefit score was calculated based on PFS. In terms of toxicity, pazopanib and nivolumab combined with ipilimumab yielded better results compared with sunitinib. Only pazopanib gained bonus points by improving the QoL. Bonus points for tail of curve, palliation and treatment-free interval were not scored in any trial. The net health benefit was 11 in pazopanib, 41.9 in nivolumab plus ipilimumab, 28.8 for PD-L1-positive patients, 22.4 for ITT population in axitinib plus avelumab, 48.7 in axitinib plus pembrolizumab, 35.2 in lenvatinib plus pembrolizumab and 50.8 in patients treated with cabozantinib plus nivolumab. The results are summarized in Table 2, and drug costs are listed in Table 3.

**Table 2:** Summary of ASCO value framework for RCC therapeutics

Treatment	Clinical benefit	Toxicity	Bonus point				Net health benefit
			Tail of Curve	Palliation	Quality of Life	Treatment-free interval	
Pazopanib	0	1	0	0	10	0	11.0
Nivolumab + Ipilimumab	37	4.9	0	0	0	0	41.9
Axitinib + Avelumab	24.8 (overall)	−2.4	0	NA	NA	0	22.4 (overall)
Axitinib+ Pembrolizumab	47	1.7	0	NA	NA	0	48.7
Lenvatinib+Pembrolizumab	34	1.2	0	NA	NA	0	35.2
Cabozantinib+Nivolumab	40	0.8	0	NA	10	0	50.8

The anticancer drugs for RCC were evaluated according to ASCO value framework, and summarized

*PD-L1* programmed death ligand-1, *NA* not available

**Table 3:** Summary of drug costs in Republic of Korea

Treatment	Total cost (dollar/4 week)
Pazopanib 800 mg qd	12,768
Nivolumab 3 m/Kg, D1 + Ipilimumab 1 mg/Kg, D1 Q2weeks for 3 months Followed by nivolumab only	39,584 (for 3 months) ➔ 10,544
Axitinib 5 mg qd + Avelumab 10 mg/Kg D1 Q3weeks	58,460
Axitinib 5 mg qd + Pembrolizumab 200 mg D1 Q3weeks	59,709
Lenvatinib 20 mg qd + Pembrolizumab 200 mg D1 Q3weeks	93,005
Cabozantinib 40 mg qd + Nivolumab 240 mg D1 Q2weeks	26,616

The drug costs for RCC were summarized

Body weight: 60 kg

### ESMO-MCBS

The value of pazopanib was evaluated using form 2c which is designed for trials with a primary endpoint other than OS or PFS or equivalence studies including non-inferiority studies. Pazopanib treatment yielded grade 4 due to improved QoL, toxicity and non-inferiority observed in PFS and OS. Nivolumab combined with ipilimumab were assessed with form 2a using HR for OS. Four trials for TKI and ICI combination were assessed using form 2b, in which treatment with axitinib plus avelumab was evaluated in ITT population. Treatment with axitinib plus avelumab and axitinib plus pembrolizumab achieved a final grade 3. Lenvatinib plus pembrolizuamb and cabozantinib plus nivolumab showed a final grade 4. Improvement in toxicity or QoL was identified in patients treated with pazopanib and nivolumab plus ipilimumab, resulting 1 level upgrade. The detailed results are summarized in Table 4.

**Table 4:** Summary of ESMO-MCBS for RCC therapeutics

Treatment	Form	Hazard Ratio^a^	Survival gain^a^ (months)	Preliminary clinical benefit grade	Early stopping or crossover ^a^	Toxicity improvement	Quality of Life improvement	Final Grade
Pazopanib	2c			4		+	+	4
Nivolumab + Ipilimumab	2a (>24mo)	0.44 (OS)	> 9 (15.2)	4		+	–	5
Axitinib + Avelumab	2b (>6mo)	0.56 (PFS)	5.4	3	–	–	NA	3
Axitinib + Pembrolizumab	2b (>6mo)	0.57 (PFS)	4	3	–	–	NA	3
Lenvatinib+Pembrolizumab	2b (>6mo)	0.39 (PFS)	14.7	3	+	–	NA	4
Cabozantinib+Nivolumab	2b (>6mo)	0.41 (PFS)	8.3	3	+	–	–	4

The anticancer drugs for RCC were evaluated according to ESMO-MCBS, and summarized

^a^Shaded area (HR and survival gain in form 2c, early stopping or crossover in form 2a) is not included in the specific forms

Hazard ratio score uses the lower limit of the interval of confidence according to guideline

*PFS* progression-free survival, *OS* overall survival, *PD-L1* programmed death ligand-1, *NA* not available

**Fig. 1:**
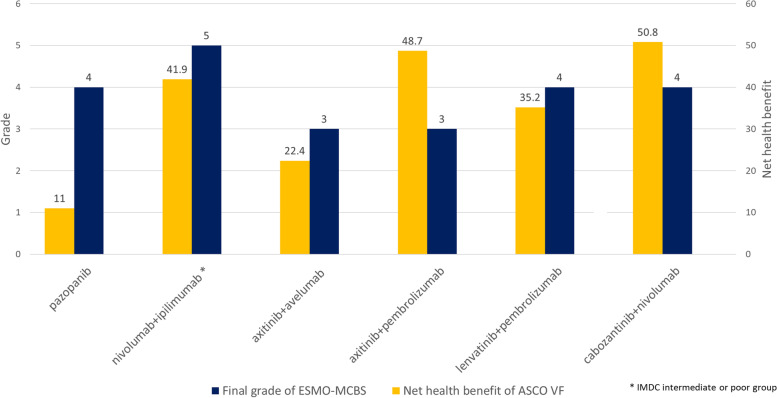
Value of emerging therapeutics in RCC. We measured value of emerging therapeutics in RCC using ASCO value framework and ESMO-MCBS

Figure 1 shows the final value of the drugs assessed by ASCO VF and ESMO-MCBS.

## Discussion

TKI including sunitinib has been the standard-of-care for metastatic RCC since mid-2000. In the past 10 years, the influx of novel medical treatments for RCC treatment has led to remarkable progress. Advances in oncology were associated with a high price tag. Therefore, RCC was accompanied by considerable economic burden not only patients but also for the health care system. At the same time, clinicians are contemplating which drugs should be administered and in what order. The value encompassing efficacy, toxicity, and cost-effectiveness has been clearly highlighted. This study reviewed the clinical application of ASCO VF and ESMO-MCBS for the novel therapeutics in RCC.

Pazopanib was approved for RCC by US FDA in 2009 [15]. The COMPARZ trial confirmed the non-inferiority of pazopanib compared with sunitinib as the first-line treatment in 2013 (median PFS 8.4 vs. 9.5 months; median OS 28.4 vs. 29.3 months) [16]. Patients receiving pazopanib manifested more adverse events such as altered hair color, weight loss, alopecia and abnormalities in liver function test, while toxicities affecting QoL, including hand foot syndrome, fatigue and grade 3 or 4 hematologic abnormality were more frequent in the sunitinib group. Among the trials included in this study, the health benefit of pazopanib according to ASCO VF was the lowest (11.0) due to the omission of clinical benefit score, and ESMO-MCBS yielded grade 4 which was the highest in form 2c.

Among the 5 trials including ICI, the trial for nivolumab and ipilimumab combination was reported first in 2018, two axitinib combination trial in 2019, and the other TKI plus ICI in 2021 [17]. The FU report including extended survival data and PRO was reported only for nivolumab and ipilimumab combination in 2019 [18]. The trial for nivolumab plus ipilimumab reported OS as a primary endpoint, while the others reported PFS as a primary endpoint, suggesting the high value of nivolumab and ipilimumab combination based on both ASCO VF (net health benefit 41.9) and ESMO-MCBS (grade 5). According to form 2a of ESMO-MCBS, grade 4 can be given in case of HR ≥ 0.7 and gain ≥9 months. Despite not-reached median OS, the FU report showed meaningful OS benefit over 9 months (HR 0.66, *p* < 0.0001). Thus, preliminary grade 4 was given to the combination of nivolumab and ipilimumab, and upgrade was done by the improvement in toxicity resulting final grade 5.

The trial of avelumab and axitinib was analyzed in two groups, including overall patients (ITT population) and patients with PD-L1-positive tumor [19]. The primary endpoints were PFS and OS in the PD-L1-positive group, and the secondary endpoint was PFS in ITT population. The US FDA approved avelumab in combination with axitinib for RCC based on PFS in ITT population. Based on form 2b of ESMO-MCBS, ITT population scored grade 3 (HR 0.69). FU data including OS or PRO has yet to be reported, which may alter the value later. Currently, avelumab is approved for Merkel cell carcinoma and urothelial carcinoma in Korea, but not for RCC.

The primary endpoints in the trial involving pembrolizumab plus axitinib were PFS and OS [20]. Significant longer PFS (HR 0.69, *p* < 0.001) and OS (HR 0.53, *p* < 0.0001) were identified in the trial involving pembrolizumab and axitinib. Due to the short FU time (median 12.8 months), the median OS has yet to be reported. ASCO VF requires only HR, while ESMO-MCBS requires both HR and absolute duration of the survival gain. Thus, the highest value was achieved with ASCO-VF (net health benefit 45.3), while ESMO-MCBS gave the final grade 3. Similar to avelumab plus axitinib, FU data would change the value of pembrolizumab and axitinib.

CLEAR and CheckMate 9ER were reported in 2021 and both trials showed improved PFS (primary endpoint) and OS (secondary endpoint) [21, 22]. They achieved upgrade in ESMO-MCBS due to early stopping. In these trials, FU data would change the value in terms of tail of curve, and QoL.

While ASCO VF and ESMO-MCBS have been used, further discussion about the appropriate group of patients for value assessment is required. The JAVELIN renal 101 trial showed differences in benefit for PD-L1-positive patients and ITT population. Occasionally, the primary and secondary endpoints are set in different group of patients, and the approval (US FDA or Korea FDA) is not always based on the primary endpoint. Sometimes, it is not clear that which group, according to endpoint or approval, is appropriate for the assessment. It was not clear whether OS was the best representative parameter. The primary goal of cancer treatment is to prolong survival, and therefore ASCO VF and ESMO-MCBS prioritized OS over PFS and gave more weight to OS. Even in the trial with OS as secondary or co-primary endpoint, the OS was used. However, the statistical design of the trial setting the endpoints is crucial and subsequent treatment may have limited benefit in OS. Further, a more recent study would gain unfavorable value. Generally, data including extended survival and PRO are reported later, thus additional points for tail of curve, palliation or QoL are unavailable. In particular, although QoL points are included in both tools, however they cannot be evaluated properly in recently reported trials. Furthermore, the additional points constitute a substantial proportion of the value. Axitinib and pembrolizumab achieved the highest value by ASCO VF (48.7) while grade 1 by ESMO-MCBS due to median OS was not reached, despite meaningful HR for death. It was also unclear whether “laboratory abnormality only” toxicity was insignificant. Is grade 4 laboratory abnormality including liver enzyme elevation, neutropenia and anemia is less important than grade 1 hair loss or fatigue? Hematologic toxicity is important in hematologic malignancy, and grade 4 neutropenia or thrombocytopenia in solid cancer is clinically significant as well. It could also reduce the quality of life due to recurrent transfusion and the risk of bleeding or infection. Thus uniform format which excludes “laboratory only toxicity” requires further discussion. Frequent errors occurred during toxicity analysis. However, the toxicity score was a relative measure of two arms, and the effect of error was marginal. Another shortcoming relates to the drug cost, which was not reflected adequately in the tools. The high cost is one of the major reasons for value assessment. ASCO VF has an entry for the cost, but it is only a reference. Further, the non-inferiority trial was not assessed appropriately. Pazopanib showed similar efficacy and a favorable toxicity profile compared with sunitinib and yielded the second highest grade [4] based on ESMO-MCBS but the lowest value (11.0) by ASCO VF. ESMO recommends the use of ESMO-MCBS for comparison of trials, while ASCO recommends against the use of VF for comparison. The discrepancy of results by the two evaluation tools may be due to the different purpose of the tools. Considering the purpose of “value evaluation of chemotherapeutics”, it is desirable to verify the results for the consistent results. These findings suggest that “similar efficacy and better toxicity” are more valuable than “better efficacy and similar toxicity”. Even allowing for the importance of PRO, the majority of oncologists appreciate the efficacy more than the toxicity. The results show that the highest score in one assessment tool does not mean the best treatment.

ASCO VF and ESMO-MCBS were applied to the new emerging therapeutics in RCC under the first-line setting and assigned the highest value cabozantinib plus nivolumab (net health benefit 49.2) and nivolumab plus ipilimumab combination (grade 5), respectively. These tools warrant further discussion and improvement. How should these assessment tools be applied in the real-world setting for approval, decisions of reimbursement or post-marketing assessment, and for patients, clinicians, decision-makers or payers (government or insurance company)? Appropriate application and revision of the tools requires in-depth discussion of experts in various fields.

## Data Availability

The datasets generated and/or analyzed during the current study are available in the figureshare repository, 10.6084/m9.figshare.18585461.v1 If additional analysis data is needed, The datasets used and/or analyzed during the current study available from the corresponding author.
